# Prediction and memory: A predictive coding account

**DOI:** 10.1016/j.pneurobio.2020.101821

**Published:** 2020-09

**Authors:** Helen C. Barron, Ryszard Auksztulewicz, Karl Friston

**Affiliations:** a Medical Research Council Brain Network Dynamics Unit, Nuffield Department of Clinical Neurosciences, University of Oxford, Mansfield Road, Oxford, OX1 3TH, UK; b Wellcome Centre for Integrative Neuroimaging, University of Oxford, FMRIB, John Radcliffe Hospital, Oxford, OX3 9DU, UK; c Max Planck Institute for Empirical Aesthetics, Frankfurt Am Main, 60322, Germany; d Department of Biomedical Sciences, City University of Hong Kong, Hong Kong; e The Wellcome Trust Centre for Neuroimaging, Institute of Neurology, University College London, London, WC1N 3BG, UK

**Keywords:** Hippocampus, Neocortex, Memory, Prediction, Interneuron

## Abstract

The hippocampus is crucial for episodic memory, but it is also involved in online prediction. Evidence suggests that a unitary hippocampal code underlies both episodic memory and predictive processing, yet within a predictive coding framework the hippocampal-neocortical interactions that accompany these two phenomena are distinct and opposing. Namely, during episodic recall, the hippocampus is thought to exert an excitatory influence on the neocortex, to reinstate activity patterns across cortical circuits. This contrasts with empirical and theoretical work on predictive processing, where descending predictions suppress prediction errors to ‘explain away’ ascending inputs via cortical inhibition. In this hypothesis piece, we attempt to dissolve this previously overlooked dialectic. We consider how the hippocampus may facilitate both prediction and memory, respectively, by inhibiting neocortical prediction errors or increasing their gain. We propose that these distinct processing modes depend upon the neuromodulatory gain (or precision) ascribed to prediction error units. Within this framework, memory recall is cast as arising from fictive prediction errors that furnish training signals to optimise generative models of the world, in the absence of sensory data.

## Highlights

• The hippocampus is involved in both memory recall and online prediction.
• Within a predictive coding framework, opposing hippocampal-neocortical interactions accompany recall and prediction.
• Here, we propose recall and prediction differentially route information within the cortical microcircuit.
• By weighting prediction errors, neuromodulation sets the dominant processing mode.
• In this framework, memory recall is cast as arising from fictive prediction errors.

## 1 Introduction

Anatomically, the hippocampus sits at the apex of a cortical processing hierarchy (Felleman and Van Essen, 1991). Inputs received by sensory cortices reach the hippocampus via the entorhinal cortex and other relay regions, which in turn, make widespread cortico-cortical connections that project the hippocampal output back to neocortex (Squire et al., 2004; Witter, 1993; Witter et al., 1989). This reciprocal anatomical connectivity equips the hippocampus with the necessary architecture to coordinate activity in neocortical circuits (Lavenex and Amaral, 2000; Vogt and Miller, 1983).

However, the functional mechanisms that underpin hippocampal-neocortical interactions remain unclear. On the one hand, the hippocampus has long been considered crucial for recall of rich and detailed memory of past episodes (Scoville and Milner, 1957; Squire and Zola-Morgan, 1991). This vivid recall of past events involves mental time travel and transient disengagement from ongoing sensorimotor experience – a process thought to be accompanied by autonoetic consciousness (Rubin et al., 2003; Tulving, 2002). This complex cognitive process can be viewed as constructive (Hassabis and Maguire, 2007; Schacter et al., 1998), where the interaction between hippocampus and neocortex mediates integration and reinstatement of information stored across modality specific cortical areas (Rugg and Vilberg, 2013; Wheeler et al., 2000). Together with the known anatomy, this supports the idea that the hippocampus indexes neocortical activity relevant to a particular memory (Marr, 1971; Teyler and DiScenna, 1986; Teyler and Rudy, 2007; Tulving and Thomson, 1973). In this manner, recall of rich and detailed memories involves a selective and facilitatory interaction between the hippocampus and neocortex.

On the other hand, the hippocampus shows a remarkable capacity to predict ongoing sensory experience in the moment (Lisman and Redish, 2009; Mehta et al., 1997; Skaggs et al., 1996; Stachenfeld et al., 2017). This predictive activity suggests the hippocampus anticipates upcoming sensory information using recent sensory inputs together with internally generated sequences that draw upon stored memories (Lisman, 1999; Pezzulo et al., 2017). Together with the known anatomy, this predictive activity suggests the hippocampus is situated high within a hierarchical *generative model.*

A generative model can be defined as an internal model that the brain can use to generate consequences of a particular action or sensory encounter. In this manner, a generative model can provide predictions for ongoing sensory experience and anticipate the consequences of a particular action before sensorimotor feedback is available. In sensorimotor control, the notion of a generative model has emerged as an important theoretical concept (Heuer and Keele, 1996; Hinton and Ghahramani, 1997; Wolpert et al., 1995). In perceptual synthesis, generative models underwrite the Bayesian brain hypothesis (Dayan et al., 1995; Doya et al., 2007; Knill and Pouget, 2004) and, in particular, predictive coding models that now dominate predictive processing accounts of perception (Bialek et al., 2001; Clark, 2013; Friston, 2005; Friston and Kiebel, 2009; Heeger, 2017; Hohwy, 2013; de Lange et al., 2018; Rao and Ballard, 1999; Spratling, 2008; Srinivasan et al., 1982; Wolpert et al., 1995).

On most accounts of predictive processing, cortical hierarchies are associated with hierarchical generative models. In this setting, higher levels are thought to generate descending predictions of lower-level (e.g. sensory) representations. These descending predictions are compared against ascending sensory representations, to form a mismatch or prediction error signal. This prediction error can be thought of as the ‘newsworthy’ information that is not predicted. As information ascends the cortical hierarchy, sensory information is therefore replaced by prediction error signals that carry the information that has yet to be explained (Clark, 2013). The prediction errors drive representations in higher levels of the cortical hierarchy to provide better predictions – and thereby suppress prediction error signals in lower levels. In addition to this online evidence accumulation (i.e., perceptual inference), prediction error signals also drive associative plasticity to update the generative model; thereby, minimising prediction errors when a similar situation is encountered in the future (i.e. perceptual learning). Finally, the relative importance or ‘newsworthiness’ of ascending prediction error signals is determined by their precision, which, as outlined below, selectively amplifies prediction error signals that convey more precise information.

To cancel or suppress the predicted component of a sensory representation, descending predictions must have a functionally inhibitory effect on neurons encoding sensory input, which at higher levels of the hierarchy corresponds to those neurons encoding prediction errors. In this manner, descending predictions – that originate in high levels of the cortical hierarchy – may be considered to resolve or ‘explain away’ ascending signals (Friston, 2005; Hinton and Ghahramani, 1997; de Lange et al., 2018). This predictive-coding framework is consistent with a large body of evidence in both humans and animals, where neuronal responses to predicted stimuli are attenuated relative to unpredicted stimuli (for example: Alink et al., 2010; Garrido et al., 2009a; Meyer and Olson, 2011; den Ouden et al., 2009). Although the precise interaction between hippocampus and neocortex during prediction remains unknown, according to the predictive coding framework, descending predictions from higher levels, such as the hippocampus, should have an inhibitory effect on neurons encoding prediction errors in lower levels of the cortical hierarchy.

This raises a potential dichotomy: as an organ of memory recall, the hippocampus excites neocortical representations to reinstate previous experience, but as an organ of prediction, the hippocampus should inhibit neocortical prediction errors. To unpack this apparent dichotomy, we first consider the functional dissociation between recall of past experience and ongoing sensory prediction. In line with previous proposals, we suggest that recall of the past and ongoing prediction rely on the same neural machinery in the hippocampus but reflect different processing modes. Using a predictive coding framework, we then characterise the hippocampal-neocortical interactions that may accompany these distinct processing modes, to consider how representations in the hippocampus exert opposing effects on neocortical circuits to instantiate both recall of rich and detailed memories and prediction of ongoing sensory experience.

In brief, we propose that during episodic recall and predictive processing distinct neocortical inhibitory interneurons differentially route information through the canonical neocortical microcircuit, to account for opposing hippocampal-neocortical interactions. Within a predictive coding framework, the inhibitory effect necessary for predictive ‘explaining-away’ may use descending inhibition, while the facilitatory effect necessary to reinstate cortical representations may be mediated by disinhibition. These opposing excitatory-inhibitory effects may be mapped onto (i) descending predictions – that *drive* inhibitory interneurons to inhibit ascending sensory input or prediction errors – and (ii) descending predictions of precision that *modulate* the gain of pyramidal neurons (or prediction error units) to reinstate cortical representations during recall. Reinstating a neocortical representation during *offline* recall of past experience may therefore be considered similar to *online* sensory processing, except that during *offline* recall the reinstated representation is ‘protected’ from ascending sensory input or prediction errors from lower hierarchical levels. Therefore, while the function of ascending prediction errors during ongoing sensory experience is to provide an *online* training signal, the function of memory recall can be cast as *offline* generation of *fictive prediction errors* that train the brain, so that it can generalize to new sensory input in the future. Below we outline the theoretical and empirical evidence that speak to this characterisation of hippocampal-neocortical interactions – and identify testable hypotheses for this model.

### 1.1 Hippocampal-neocortical interactions during memory recall

The hippocampus plays a crucial role in the recall of rich and detailed memories of past experience, otherwise termed episodic memories. This is evident in the dramatic amnesia observed in patients with bilateral hippocampal lesions (Scoville and Milner, 1957). In these patients, remote memory appears to be spared, leading to the suggestion that recall of remote episodic memory may be hippocampal-independent (Standard Model of Memory Consolidation) (McClelland et al., 1995; Squire and Alvarez, 1995). However, alternative models (such as Multiple Trace Theory) argue that the hippocampus is required for recall of rich and detailed memory in perpetuity (Nadel and Moscovitch, 1997, 1998). While empirical support for these competing theories has been discussed in detail elsewhere (Barry and Maguire, 2019; Frankland and Bontempi, 2005; Nadel and Hardt, 2011), here we distil the common ingredients that describe hippocampal-neocortical interactions during memory recall. Notably, these dominant theories agree that during recall of rich and detailed memory, the hippocampus mediates neocortical memory reinstatement, if only temporarily.

**Fig. 1:**
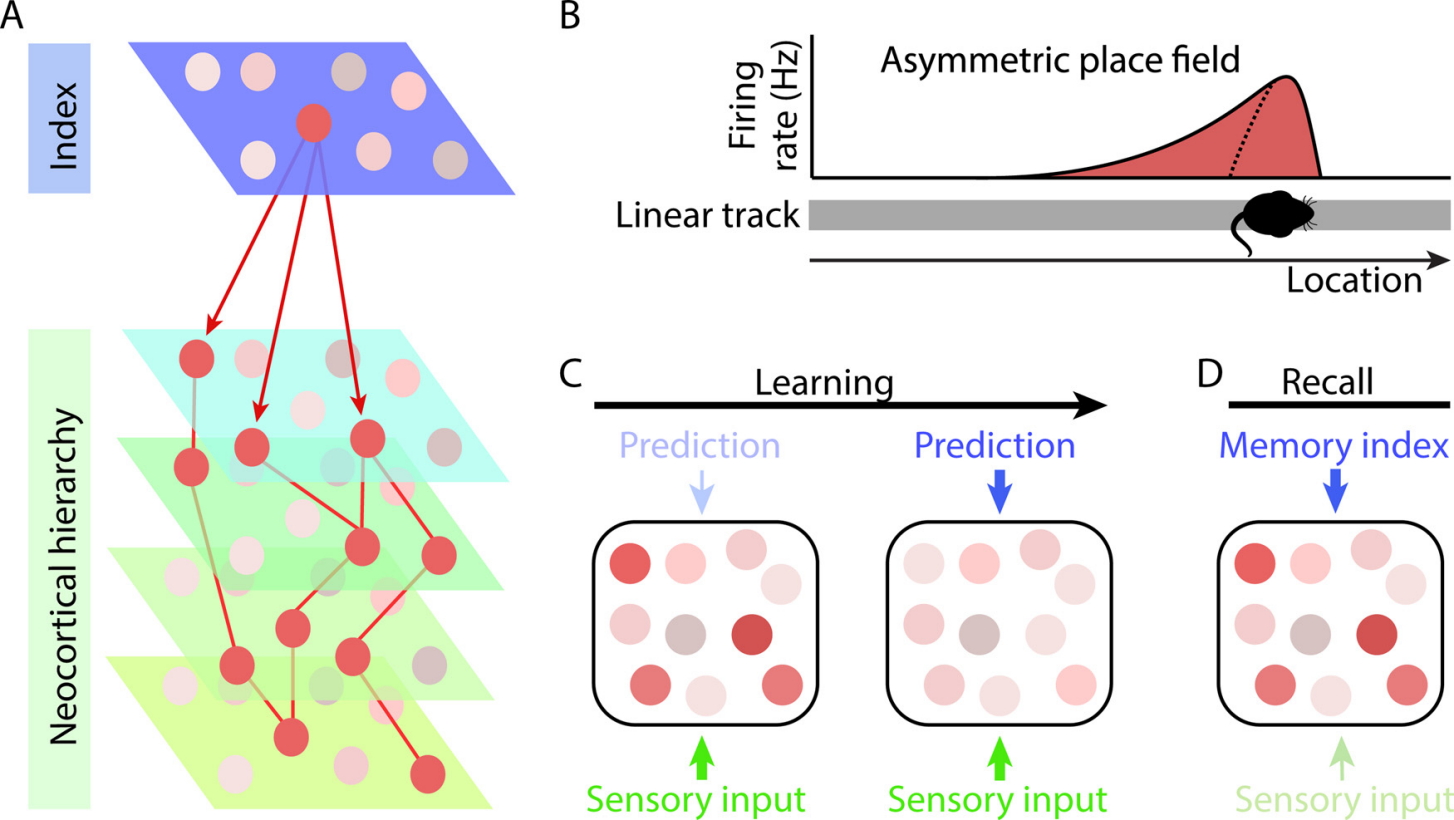
The hippocampus as both a memory index and a generative model. **A)** Schematic illustrating the hippocampus as a memory index: During memory recall, activity patterns across neocortex are reinstated to recapitulate previous sensory experience (shown in red, distributed across the neocortical hierarchy). The hippocampus (shown in blue), which is anatomically situated at the top of a cortical processing hierarchy, is thought to orchestrate this reinstatement by binding and linking activity patterns stored across distributed neocortical networks. **B)** When rodents repeatedly navigate on a one-dimensional track (shown in grey), spatially tuned principal cells in the hippocampus (shown in red) show a backward skew in their firing rate (filled line) relative to the first run on the linear track (dotted line) (schematic adapted from Mehta et al., 1997). This backward skew can be explained by a Successor Representation (Stachenfeld et al., 2017) where the hippocampus represents upcoming locations or states that are reliably predicted from the current location or state. **C–D)** Schematic showing neocortex at an intermediary level in the cortical hierarchy. Within a predictive coding framework, the dual aspect role of the hippocampus gives rise to two complementary hippocampal-neocortical interactions. Descending inputs from the hippocampus are shown in blue. An example subset of cells in the neocortex are shown in the black box with low firing rate indicated in pale pink and high firing rate indicated in red. Ascending sensory input (or prediction error signals) are shown in green. **C)** As a generator of predictions, or generative model, the hippocampus accumulates ascending prediction errors from neocortical neurons lower in the hierarchy (not shown) and responds with descending predictions to neocortex that inhibit the neocortical prediction error signals. Left-hand panel: When the sensory input is unexpected, the resulting prediction errors are represented in the neocortical hierarchy. Right-hand panel: With learning, the hippocampal generative model is updated until the hippocampal predictions ‘explain away’ prediction errors by suppressing neocortical activity. **D)** As a memory index, the hippocampus provides descending input to the neocortex to selectively reinstate activity patterns that recapitulate previous sensory experience. The hippocampal memory index can thus facilitate neocortical activity, even in the absence of sensory input.

Memory recall can be defined as vivid recollection of past events, a process that involves mental time travel and imagery with transient disengagement from ongoing sensorimotor experience (Rubin et al., 2003; Tulving, 2002). Behaviourally, recall of past experience may provide a means to simulate and evaluate the hypothetical consequences of future decisions (Atance and O’Neill, 2001; Schacter et al., 2007; Szpunar, 2010), reducing the uncertainty inherent in deliberative behaviour. At the physiological level, memory recall is accompanied by reactivation of distributed activity patterns evinced during an original experience (McClelland et al., 1995). Mechanistically, this is thought to be achieved via interactions that span a neocortical-hippocampal-neocortical loop. Thus, during sensory experience, neocortical activity patterns are passed up the cortical hierarchy to the hippocampus (Merzenich et al., 1990), and during memory recall this interaction is thought to be inverted – with the hippocampus facilitating and coordinating activity, across distributed neocortical circuits (Fig. 1A). Data from both humans and rodents provide support for this view. For example, neocortical memory reinstatement can be observed in humans using functional Magnetic Resonance Imaging (fMRI) to decode memory-specific activity patterns (Baldassano et al., 2017; Chadwick et al., 2010; Staresina et al., 2012). Moreover, fluctuations in the hippocampal Blood Oxygen Level Dependent (BOLD) signal predict trial-by-trial measures of neocortical activity patterns (Bosch et al., 2014), suggesting evidence for a coordinated faciliatory interaction between hippocampus and neocortex during recall.

Optogenetic manipulations in rodents further corroborate this picture. When memory-specific neurons in hippocampus are tagged and selectively silenced in a contextual fear-conditioning paradigm, reinstatement of neocortical memory traces and behavioural memory expression are impaired (Cowansage et al., 2014; Tanaka et al., 2014). However, even when the hippocampus is silenced, memory impairment can be mitigated if the neocortical memory trace is activated using optogenetic stimulation (Cowansage et al., 2014). This suggests that during recall, memory-specific neurons in the hippocampus reinstate selective representations in neocortex.

Together these findings support a model whereby the hippocampus represents an ‘index’ (Teyler and DiScenna, 1985, 1986; Teyler and Rudy, 2007) or ‘summary sketch’ of the neocortical representation. A sparse activity pattern in the hippocampus may represent the specific conjunction or combination of representations in neocortex that together give rise to the full activity pattern (McClelland et al., 1995). Therefore, even if the hippocampus receives an incomplete version of the activity pattern, the hippocampus can “pattern complete” to facilitate reinstatement of the entire activity pattern across neocortex (Rolls, 2013).

Physiologically, this hippocampal index may be attributed to pyramidal cells in the CA1 and CA3 regions of the hippocampus. Pyramidal cells in CA1 and CA3 are known to represent space (O’Keefe, 1976) and time (MacDonald et al., 2011; Pastalkova et al., 2008), but also contextual information (McKenzie et al., 2014). Together these ‘where’ and ‘when’ representations may constitute a neural code that describes the statistical regularities of space and time, capturing variance along two principal dimensions of everyday experience (Eichenbaum, 2017; Friston and Buzsáki, 2016; McKenzie et al., 2014; Schiller et al., 2015; Whittington et al., 2019). In addition, the contextual ‘what’ component of these hippocampal codes allows for translational invariance across space and time, to endow narratives with a particular kind of content. Pyramidal cells in the hippocampus thus provide the necessary building blocks for representing rich and detailed experience, either of the lived world, or of the past.

### 1.2 Predictive activity in the hippocampus

While the hippocampus is necessary for recall of episodic memories, the anatomical and functional architecture of the hippocampus suggests a cardinal role in the handling of abstract, high-level prediction errors. This can be seen in terms of its role as a comparator network: predictive sequences in CA3 are sent to area CA1 and compared with a second major input that conveys sensory information from neocortex (Hasselmo and Wyble, 1997; Lisman and Grace, 2005; Penny et al., 2013; Vinogradova, 1975, 2001). This comparator circuitry may account for predictive (‘match’) signals in the hippocampus (Brown and Aggleton, 2001; Hannula and Ranganath, 2008; Preston and Gabrieli, 2008), but also prediction error (‘mismatch’) signals that may mediate rapid novelty detection in both humans and animals (Chen et al., 2011; Duncan et al., 2012; Fyhn et al., 2002; Jenkins et al., 2004; Kumaran and Maguire, 2006; Ruusuvirta et al., 1995).

During ongoing sensory experience, predictive activity can be observed in the hippocampus in the form of phase precession (O’Keefe and Recce, 1993; Skaggs et al., 1996). Phase precession is a phenomenon where the phase at which CA1 pyramidal cells fire in the theta rhythm advances as an animal moves through the cells’ preferred place fields. In this manner, pyramidal cells show cued prediction of the sequence of upcoming positions (Jensen and Lisman, 1996; Tsodyks et al., 1996). This predictive activity is thought to be generated using incoming sensory information together with stored memory sequences in the CA3 region of the hippocampus (Lisman, 1999). At a choice point in a maze, where animals pause and show searching behaviour termed vicarious trial and error, this predictive activity can manifest as non-local activity that sweeps through the successive locations in the maze, thus spanning future possible trajectories (Johnson and Redish, 2007). Hippocampal phase precession can also be observed during theta oscillations in the non-spatial domain, during the delay period of a memory task, where environmental cues are kept constant (Pastalkova et al., 2008), or when a rat is removed from a ledge and required to jump to safety to avoid a shock (Lenck-Santini et al., 2008).

A related, but likely independent, phenomenon is experience-dependent asymmetric expansion of place fields (Blum and Abbott, 1996; Mehta et al., 1997). This can be observed when rodents repeatedly travel in a particular direction along a linear track. With experience of the track, hippocampal pyramidal cells start to fire before the animal visits the preferred spatial location of each cell. This anticipatory activity manifests as a backward skew in the spatial firing fields of pyramidal cells (Fig. 1B), which can be described computationally by the successor representation (Stachenfeld et al., 2017), and other generative models based on Markovian processes, where the probability of a future event depends only on the present state (Chen et al., 2014; Kaplan and Friston, 2018). Rather than representing explicit spatial or temporal information, the successor representation encodes the states of the environment in terms of their predictive relationships with other states, thus providing an efficient estimate of long-term future reward (Dayan, 1993; Gershman, 2018). In humans, the successor representation can explain the hippocampal BOLD signal, even when participants navigate through an abstract set of discrete stimulus associations (Garvert et al., 2017).

Here, we focus on hippocampal predictions that occur during *ongoing sensory experience*. However, predictive activity has also been reported in the hippocampus during ‘offline’ periods of rest or sleep, which can predict forthcoming behaviour in a manner consistent with model-based sequential planning (Pfeiffer and Foster, 2013; Singer et al., 2013) or ‘preplay’ of future spatial trajectories (Dragoi and Tonegawa, 2011, 2013). These different types of predictive activity, ‘online’ versus ‘offline’, may reflect different processing modes in the hippocampus, characterised by distinct oscillatory patterns in the hippocampal local field potential that are dominated by a theta rhythm and sharp-wave ripple (SWR) events, respectively. Unlike, predictive activity during ongoing sensory experience, we propose that hippocampal-neocortical interactions that accompany ‘offline’ predictive activity may be analogous to interactions observed during recall of past experience and thus described as episodic simulation (Schacter et al., 2007). For the remainder of the article, we restrict the term *prediction* to refer to predictions of ongoing sensory experience, where the interaction between the hippocampus and neocortex may be interpreted within a predictive coding framework (as opposed to a simulation framework). Similarly, we reserve *recall* for mnemonic processes that are not contingent on current sensory input. These two processes, recall and prediction, may be considered two different aspects of ‘pattern completion’, and differ from new learning driven by prediction error signals (analogous to ‘pattern separation’, Yassa and Stark, 2011). However, as discussed below, within a predictive coding framework, recall may be framed as a fictive prediction error.

### 1.3 Hippocampal-neocortical interactions during prediction

While the hippocampal-neocortical interactions that accompany recall are reasonably well established, the precise interactions that accompany prediction of ongoing sensory experience are less clear. To account for hippocampal-neocortical interactions during prediction, we appeal to theoretical and empirical work on predictive processing. From a predictive-coding perspective, and in virtue of its connections with the neocortex, the hippocampus can be regarded as a hub or centre of a deep or hierarchical generative model that reaches all the way out to sensory cortex (Liu et al., 2018; Mesulam, 2013). On this view, the hippocampus can be considered to play a central role in hierarchical predictive coding – a formulation of recurrent neuronal message passing, informed largely by studies of visual processing (Bastos et al., 2012; Heeger, 2017; Kok et al., 2012a, 2012b; Rao and Ballard, 1999; Shipp, 2016; Spratling, 2010). However, rather than generating predictions in a particular sensory modality, predictions generated in the hippocampus may underwrite multisensory integration (Kok and Turk-Browne, 2018), and furnish predictions that call on multimodal representations (Barron et al., 2013). In addition to this domain general aspect, predictive activity in the hippocampus should be endowed with a temporal depth. This follows because higher levels of the predictive coding hierarchy accumulate evidence for representations at progressively longer temporal scales (Kiebel et al., 2008). In other words, by being positioned at the apex of the cortical hierarchy, the hippocampus may generate multisensory predictions (i.e., ‘what’) with an ordinal aspect (i.e., ‘when’), because it accumulates evidence for trajectories or narratives that have a deeper reach into the future (and past).

Central to the predictive-coding formulation is the idea that the brain actively predicts upcoming sensory experience, to reduce or ‘explain away’ activity in lower-level areas. This provides an efficient processing hierarchy, where at each level only the discrepancy between the sensory input and the predictions received from higher-level brain areas are represented, as a prediction error signal (Bastos et al., 2012; Rao and Ballard, 1999; Shipp, 2016). Evidence from single-unit recordings in macaque inferotemporal cortex support this view, showing reduced responses to predicted sequences of natural images, when compared to unpredicted sequences (Meyer and Olson, 2011). Similarly, human imaging studies show reduced responses in sensory neocortex for predictable compared to unpredictable or deviant stimuli (Alink et al., 2010; Garrido et al., 2009a; Ouden et al., 2010; Todorovic et al., 2011), which cannot be accounted for by attentional effects (Alink et al., 2010).

However, while studies in both humans and non-human primates show robust evidence for reduced neural responses to predicted stimuli, this does not necessarily imply an inhibitory predictive signal. To reveal the precise mechanism that accounts for reduced neural responses to predicted stimuli, researchers have taken advantage of genetic tools available in mice. For example, by combining electrophysiology, calcium imaging and optogenetic manipulations, the attenuation of stimulus-evoked responses in excitatory cells of auditory cortex – during ongoing movement – can be attributed to postsynaptic inhibition (Schneider et al., 2014). This inhibition implicates local parvalbumin-positive (PV+) interneurons, which receive excitatory signals from secondary motor cortex analogous to a corollary discharge; i.e., a prediction of sensory consequences (Schneider et al., 2014). In line with the interaction between secondary motor cortex and auditory cortex (Schneider et al., 2014), and the tenets of predictive coding (Jehee and Ballard, 2009; Rao and Ballard, 1999), this kind of result suggests that predictions that derive from higher-order brain regions, such as the hippocampus, should exert an inhibitory effect on neocortex, reducing sensory-bound responses. Indeed, in primary visual cortex (V1), experience-dependent changes in descending projections from the retrosplenial cortex – a brain region that receives input directly from the hippocampus – are thought to inhibit ascending sensory input (Makino and Komiyama, 2015).

While there are countless examples to suggest that predictions reduce neural activity^1^, the predictive-coding model has been challenged by evidence suggesting predictions can enhance rather than reduce neural activity (Chaumon et al., 2008; Doherty et al., 2005; Hindy et al., 2016). The usual explanation – for these seemingly contradictory findings – is that attention, which is known to enhance neural activity, effectively opposes the inhibitory effect of prediction by enhancing the precision of prediction error units (Auksztulewicz and Friston, 2015; Chennu et al., 2016; Smout et al., 2019). In line with this framework, fMRI measurements in humans show that attention boosts the neural responses to sensory evidence, such that it reverses the inhibitory effect of prediction (Kok et al., 2012a). Similarly, the precision of prediction errors is thought to gradually increase with learning (Friston, 2008; Garrido et al., 2009b; Moran et al., 2013): intuitively, in a familiar environment, even minor deviations from perceptual predictions may be deemed as "newsworthy". This speaks to the importance of precision or gain control in the mediation of enhanced neuronal responses.

### 1.4 Reinstatement or explaining away?

The hippocampus may thus be considered to have two cardinal functions, that involve episodic recall and prediction of ongoing experience. While this dual use of memory likely relies upon the same neural machinery within the hippocampus, from a predictive coding perspective recall and prediction should give rise to opposing interactions between the hippocampus and neocortex. Namely, during memory recall the hippocampus should selectively facilitate neocortical representations – by increasing the excitability of appropriate prediction error units – to reinstate previous experience. Conversely, during predictive coding, the hippocampus should inhibit prediction error units that are reporting unexplained sensory inputs.

Although it remains to be seen whether the *same* cells in hippocampus generate predictions and support memory recall, evidence from rodents shows that these two functions are likely supported by the same hippocampal cell-type; namely, pyramidal cells. Indeed, prediction can be considered the necessary consequence of a conjunctive or relational code that formalises both spatial and abstract representations supported by hippocampal pyramidal cells (Eichenbaum, 2004). This means that during prediction, the hippocampus may be considered to hold a pointer to neocortical representations of a predicted sensory cue or state, directly analogous to a memory index.

However, if hippocampal cells provide a memory index *for representations* in sensory neocortex, while also generating predictions *of those representations*, what does this tell us about the interaction between the hippocampus and neocortex? Crucially, characterising the function of the hippocampus as a memory index that reactivates representations in neocortex is fundamentally at odds with the idea that the hippocampus provides descending predictions to explain away prediction errors at lower cortical levels. To spell out this apparent contradiction: within a predictive coding framework the role of a memory index is to *increase* neural activity to select a particular cortical representation, while the role of a prediction is to *decrease* cortical activity (Fig. 1C–D).

To reconcile this apparent contradiction, we appeal to a dual role of descending predictions in predictive coding to characterise the synaptic interactions that may underlie communication between the hippocampus and neocortex. We propose that the indexing or selection of cortical representations involves changing the relative influence of descending predictions from the hippocampus and ascending signals from lower cortical regions. This is achieved by increasing the *precision* of prediction errors in hippocampal targets. In predictive coding, precision describes the reliability or confidence ascribed to prediction errors at each level of the hierarchy. Heuristically, precision modulates the gain of ascending prediction errors to convey ‘newsworthy’ information; namely, things that were not predicted but are *predictable* (Clark, 2013). Therefore, in a noisy or volatile environment, precision at lower levels is reduced, so that sensory prediction errors are effectively ignored by reducing their influence on belief updating at higher levels. Conversely, increasing the precision of high-level prediction errors protects top-down predictions from revision by bottom-up prediction errors from lower levels. It is this mechanism we associate with the selection of high-level cortical representations by the hippocampus.

In engineering, predictive coding is known as Kalman filtering, where precision corresponds to the Kalman gain that controls the influence of prediction errors on state estimation. Psychologically, precision is associated with attentional gain or selection (Feldman and Friston, 2010). Physiologically, this gain control is thought to be mediated by disinhibiting neocortical superficial pyramidal cells that are thought to signal prediction errors (Bastos et al., 2012). Crucially, this disinhibition may involve fast synchronous interactions between superficial pyramidal cells and fast spiking inhibitory interneurons (Auksztulewicz and Friston, 2015; Fries, 2005; Kann et al., 2014; Sohal et al., 2009).

Here, we build on this proposal, to suggest that the effects of descending hippocampal projections – and subsequent processing throughout the cortical hierarchy – are determined by two factors: the dynamic mode of the hippocampus, and the subsequent routing of signals within the cortical microcircuit. In short, the dynamic mode of the hippocampus can be characterised by hippocampal oscillations, which are dominated by the theta rhythm during ongoing sensory prediction in rodents (Pezzulo et al., 2017), and by sharp-wave ripple (SWR) events during episodic memory recall in humans (Norman et al., 2019; Wimmer et al., 2019). We propose the hippocampal oscillatory state sets the dynamic mode for hippocampal-neocortical communication. The effect of activity generated by the hippocampus is then determined by the precise routing within the neocortical microcircuit. During prediction, descending projections are mediated by direct inhibition of ascending cortical prediction errors encoded by cortical pyramidal cells, while recall is facilitated by modulatory disinhibition of the same cortical cells. Thus, one can map inhibition and disinhibition of cortical pyramidal cells onto two cardinal components of predictive coding, namely *first-order predictions* (of content) and *second-order predictions* (of precision) – as described in the context of visual processing (Kanai et al., 2015; Shipp, 2016). Below, we examine the empirical evidence in favour of these distinct mechanisms.

### 1.5 Inhibition versus disinhibition

A core feature of neocortex is its layered structure (Felleman and Van Essen, 1991). Pyramidal neurons in layer 2/3 receive both bottom-up sensory information from excitatory neurons in layer 4 and top-down inputs at their distal dendrites in superficial layer 1 (Zhang et al., 2014). Plasticity in layer 1 may therefore allow for dynamic changes to the weighting of descending relative to ascending inputs (Abs et al., 2018; Letzkus et al., 2015). In predictive coding descending predictions from higher cortical areas (here, ultimately from the hippocampus) are proposed to provide top-down inputs that suppress activity in lower areas of the cortical hierarchy, particularly in superficial layers (Bastos et al., 2012). Within this framework, descending projections that convey predictions must therefore either be inhibitory (e.g., long-range GABAergic projections) or target local inhibitory interneurons (e.g., in superficial cortical layers).

Although extrinsic corticocortical and allocortical-neocortical connections are predominantly excitatory (i.e., glutamatergic), and inhibition is mostly locally sourced in neocortex, mounting evidence suggests interregional long-range GABAergic connectivity is more prevalent than previously assumed (Caputi et al., 2013), including projections that originate in hippocampus and target neocortex. While the functional significance of these long-range GABAergic projections remains unclear, one intriguing possibility is that they carry predictions from the hippocampus to neocortical brain regions. The anatomical profile of long-range projecting GABAergic cells is consistent with this view; where anatomical, molecular and electrophysiological approaches have revealed long-range GABAergic connections from hippocampus to entorhinal cortex (Melzer et al., 2012) and to the retrosplenial cortex (Ferreira-Fernandes et al., 2019; Jinno et al., 2007; Miyashita and Rockland, 2007; Yamawaki et al., 2019a), notably two brain regions that provide a crucial gateway between the hippocampus and other cortical regions (Kaboodvand et al., 2018; Witter, 1993).

The postsynaptic target of these long-range projecting GABAergic cells may depend on the precise target region, with reported evidence for preferential targeting of both pyramidal neurons (Jinno et al., 2007; Yamawaki et al., 2019a) and inhibitory interneurons (Melzer et al., 2012). Long range GABAergic projections from CA1 to retrosplenial cortex are reported to target apical dendrites in layer 1 of pyramidal neurons in deep layers (layer 5) (Yamawaki et al., 2019a). Interestingly, long-range projecting GABAergic cells originating in the hippocampus have larger axon diameter and a thicker myelin sheet than equivalent CA1 pyramidal cells connecting to the same region (Jinno et al., 2007). This suggests that inhibition deriving from hippocampus arrives before excitatory afferents, providing the necessary properties for an inhibitory hippocampal-neocortical interaction with efficient temporal synchronisation across hippocampal-neocortical circuits (Buzsáki and Chrobak, 1995). However, despite detailed neurochemical and anatomical characterisation of long-range projecting GABAergic neurons (Jinno, 2009), their functional significance remains to be established. Future investigations are necessary to determine whether these projections carry descending predictions to ‘explain away’ activity in lower-level regions of the cortical hierarchy.

Alternatively, hippocampal predictions may instantiate neocortical inhibition by locally sourced neocortical inhibitory cells, targeted by long-range excitatory projections. A suitable candidate population of inhibitory cells are found in superficial cortical layers (Abs et al., 2018; Anderson and Martin, 2006; Shipp, 2007; Yamawaki et al., 2019b). Simultaneous whole-cell patch-clamp recordings show that inhibitory interneurons in layer 1 provide strong monosynaptic inhibition to layer 2/3 pyramidal cells, whose apical dendrites project to superficial layers (Chu et al., 2003; Wozny and Williams, 2011). Furthermore, stimulation of layer 1 barrel cortex in the rat results in powerful inhibitory effects on whisker-evoked responses (Shlosberg et al., 2006). Inhibitory cells in superficial cortical layers therefore constitute a suitable candidate for suppressing predicted neocortical activity by targeting pyramidal cells that represent prediction errors. In short, one possibility is that inhibitory cells in superficial layers of neocortex receive descending, first-order predictions from regions that reside at the apex of the cortical processing hierarchy (Fig. 3).

Evidence to support this architecture is beginning to emerge. For example, in superficial layers of primary visual cortex experience-dependent changes in top-down inputs that derive from the retrosplenial cortex are gated by somatostatin positive (SOM+) interneurons (Makino and Komiyama, 2015). In turn, retrosplenial cortex receives hippocampal projections that terminate in superficial layers (Sugar et al., 2011). A similar configuration is seen in somatosensory cortex, where apical dendrites in layer 1 receive descending projections from deep layers of the perirhinal cortex – the final outpost of the medial-temporal loop (Doron et al., 2019). A notable exception to the rule can be observed in entorhinal cortex, where projections from the hippocampus terminate in deep layers (V or VI) (Burwell and Amaral, 1998; Suzuki and Amaral, 1994). While the entorhinal cortex may play a unique role at the interface between hippocampus and neocortex, beyond the medial-temporal lobe current empirical evidence suggests inputs that derive from hippocampus target locally sources neocortical inhibitory cells that reside in superficial cortical layers.

Notably, the local cortical circuit motif that facilitates direct inhibition of ascending signals also has the capacity to mediate disinhibition – that is inhibition of inhibition. Disinhibition provides a mechanism to counter the inhibitory effect of descending first order predictions. Therefore, selective disinhibition can effectively reduce inhibition onto a target population to selectively increase its expression, enabling particular cortical dynamics (and recurrent cortico-hippocampal exchanges) that would look exactly like hippocampal ‘indexing’. Compared to other mechanisms that increase excitatory drive to the target population via multiplicative or additive modulation of excitation (Carrasco, 2011; Reynolds and Heeger, 2009), or via competitive inhibition (see Sridharan and Knudsen, 2015 for theoretical comparison of these mechanisms), disinhibitory mechanisms readily account for enhanced processing of a representation *in the absence of sensory input*. Relative to mechanisms that involve competitive inhibition, disinhibition releases cortical activity patterns in a manner that is independent of competing representations (or distractor representations, in the context of attentional mechanisms (Sridharan and Knudsen, 2015)). By isolating the neocortical target in this manner, correlated noise between the target and competing representations is reduced.

**Fig. 2:**
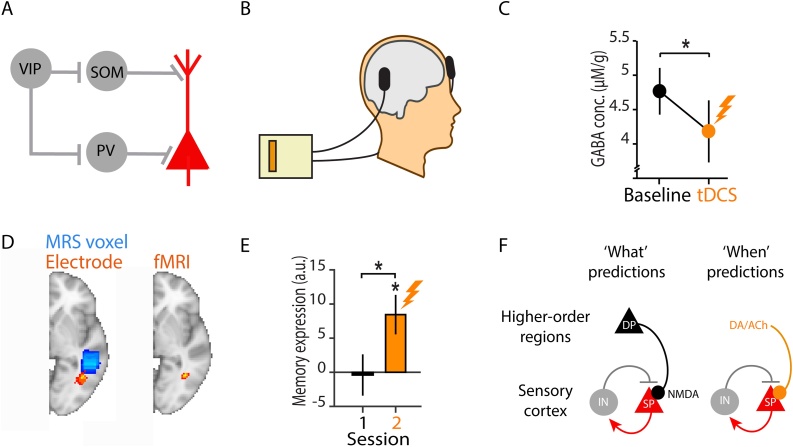
Inhibition and disinhibition within the canonical neocortical circuit motif. **A)** Schematic showing a circuit motif that employs disinhibition (i.e. inhibition of inhibition). Typically, VIP+ interneurons provide disinhibitory control by targeting PV+ and/or SOM+ interneurons that otherwise inhibit the target excitatory principal neurons. VIP, PV and SOM refer to VIP+, PV+ and SOM+ respectively. Interneurons are shown in grey. Pyramidal cells are shown in red. **B–E)** Disinhibition of human neocortex leads to re-expression of associative memories formed between visual stimuli that are rotationally invariant. Furthermore, for two overlapping memories, disinhibition in human neocortex increases memory interference. Adapted from Koolschijn et al., 2019. **B)** Schematic showing how transient disinhibition of human neocortex can be achieved using unilateral anodal transcranial direct current stimulation (tDCS), with the anodal electrode positioned above a target region, the anterior lateral occipital cortex (LOC), which has previously been shown to encode associations between visual stimuli that are rotationally invariant (Barron et al., 2016). The cathodal electrode was positioned over the contralateral supraorbital ridge. **C)** When tDCS is applied for 20 minutes using the configuration shown in *B*, a reduction in the concentration of neocortical GABA is observed in anterior LOC, measured with Magnetic Resonance Spectroscopy (MRS). **D)** Left: average position of the anodal tDCS electrode, projected into the brain (red-yellow, with group average in yellow) and average position of the MRS voxel (blue) from which the change in concentration of GABA was measured. Right: when neocortical GABA is reduced using brain stimulation, functional Magnetic Resonance Imaging (fMRI) reveals re-expression of associative memories and an increase in memory interference in the brain region underneath the anodal electrode. **E)** Underneath the anodal electrode, an increase in associative memory expression measured with fMRI can be observed during application of tDCS (providing effective disinhibition), suggesting that expression of associative memories is otherwise quenched by cortical inhibition. **F)** Prediction signalling in different domains affects the gain in sensory cortical regions, expressed as interactions between ensembles of superficial pyramidal cells (SP) and inhibitory interneurons (IN). However, the exact neuromodulatory mechanisms are domain-specific: ‘what’-predictions are mediated by NMDAR-dependent short-term plasticity contingent on the postsynaptic effects of descending connections from deep pyramidal cells (DP) of higher-order regions, such as the hippocampus, on SP of lower-order regions; ‘when’-predictions are instead subserved by classical (e.g., dopaminergic, DA, or cholinergic, ACh) modulation of postsynaptic gain in lower-order sensory regions. When interacting together, these temporal predictions could be specific to a particular stimulus content (Auksztulewicz et al., 2018).

Evidence for circuit motifs that employ disinhibition have been identified across several cortical regions (Letzkus et al., 2015). Typically, vasoactive intestinal peptide positive (VIP+) interneurons are thought to provide disinhibitory control, by targeting parvalbumin positive (PV+) and/or somatostatin positive (SOM+) interneurons that otherwise inhibit target excitatory neurons (Pfeffer et al., 2013; Pi et al., 2013) (Fig. 2A). Compared to other interneuron subtypes (PV+ and SOM+), VIP+ interneurons receive the largest proportion of cortical input, with distal cortical inputs projecting from deep cortical layers (Wall et al., 2016). This suggests that VIP+ interneurons are well positioned to implement the effect of descending projections. In addition to VIP+ interneurons, layer 1 interneurons positive for neuron-derived neurotrophic factor (NDNF) have also been implicated in experience-dependent disinhibition (Abs et al., 2018).

**Fig. 3:**
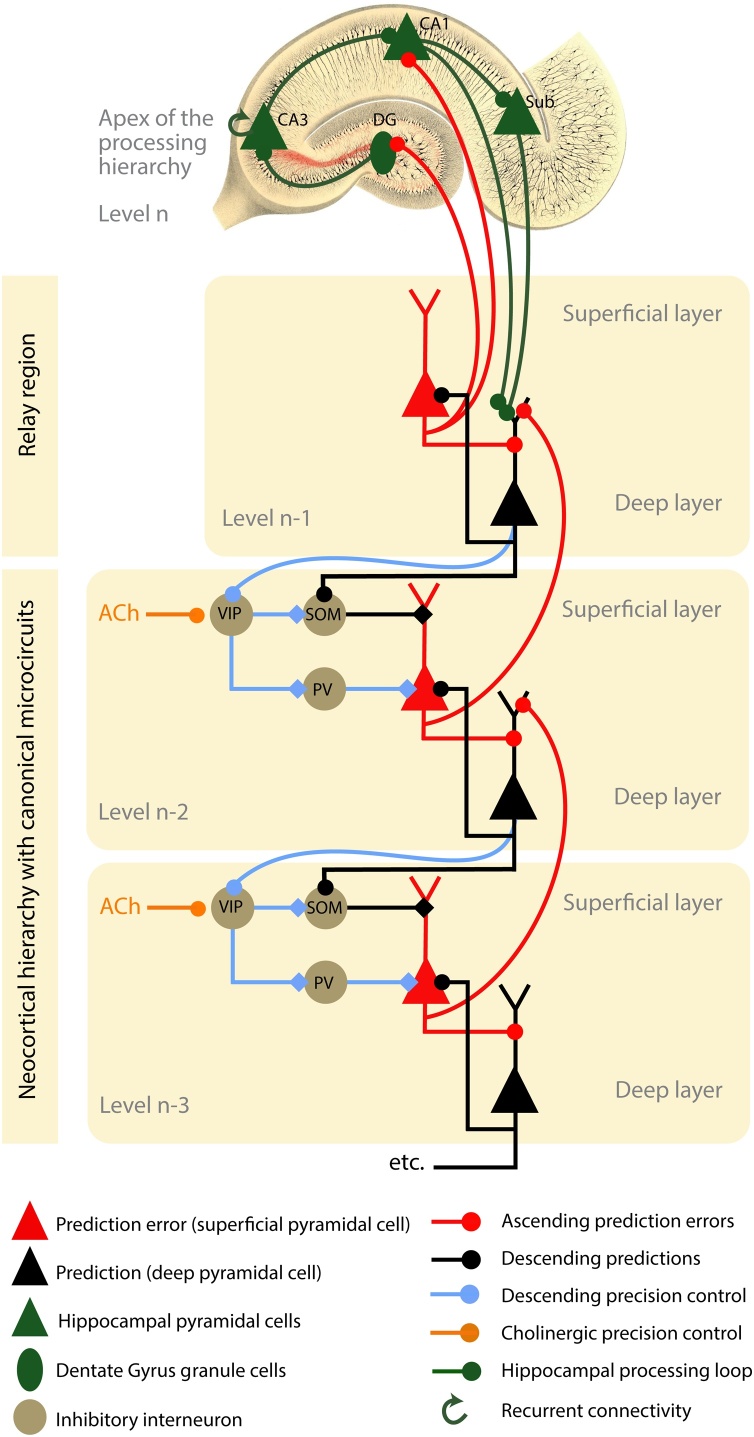
Schematic showing the proposed neuronal architecture underlying inhibitory and facilitatory hippocampal-neocortical interactions. Within the neocortical hierarchy, message passing is orchestrated by a canonical microcircuit that includes both excitatory (red and black) and inhibitory (beige) cells. In the superficial layers of each cortical level, superficial pyramidal cells (red) compare the activity of representational units (black) with top-down predictions relayed via SOM+ inhibitory interneurons (SOM). These interneurons are targeted by descending prediction signals that originate in deep pyramidal cells (black) from the level above. The mismatch between representations and descending predictions (black lines) constitutes a prediction error. This prediction error signal (red lines) is passed back up the cortical hierarchy and is received by prediction units (black) that drive responses in higher representational units, or, at the apex of the processing hierarchy, in the hippocampus. Therefore, as information moves up the cortical processing hierarchy, sensory input is replaced by prediction error signals that convey the only information yet to be explained. These prediction error signals drive representations in higher levels of the cortical hierarchy to provide better predictions, but also drive associative plasticity to update internally generated predictions that in the hippocampus draw on memory. The output from the hippocampus targets neocortex via glutamatergic projections to deep pyramidal cells (black, e.g. in the entorhinal cortex), or via long-range GABAergic projections to superficial cells (e.g. in retrosplenial cortex Yamawaki et al., 2019a; not shown here). Using a predictive coding framework, we propose that the hippocampus uses a unitary code with a dual aspect function. This dual aspect function can be characterised as follows: During prediction, the hippocampus can provide multi-sensory predictions to ‘explain away’ prediction errors at lower levels of the cortical hierarchy. This manifests as an inhibitory hippocampal-neocortical interaction – here mediated by SOM+ inhibitory interneurons. During memory recall, the hippocampus can provide a memory index to neocortex, to selectively reinstate activity patterns across distributed neocortical networks, which manifests as a facilitatory hippocampal-neocortical interaction – here mediated polysynaptically via VIP+ and SOM+/PV+ inhibitory interneurons. We propose that the diversity of inhibitory interneurons – and their selective responses to classical neuromodulators or NMDAR-mediated stimulation– provide the necessary machinery for complementary inhibitory and facilitatory hippocampal-neocortical interactions. Computationally, the facilitatory (disinhibitory) effect of hippocampal projections would, in this scheme, encode the precision of prediction error units by modulating their postsynaptic excitability. For simplicity, we have omitted many connections and cell types in the canonical microcircuit (e.g., spiny stellate cells in layer 4) and in the hippocampus. Furthermore, we have omitted descending projections directly to PV+ interneurons. Excitatory synapses are denoted with lines ending in a circle, while inhibitory synapses are denoted by a diamond. Note that superficial pyramidal cells receive excitatory and inhibitory influences that underwrite a prediction error, while the precision of the encoded prediction error is controlled by modulatory (orange) interactions with VIP+ inhibitory interneurons. ACh refers to acetylcholine. PV, SOM and VIP refer to PV+, SOM+ and VIP+ interneurons. DG refers to the dentate gyrus, Sub refers to subiculum, which together with CA1 and CA3 constitute subfields of the hippocampus that reside along the performant pathway; ‘n’ refers to the level in the cortical hierarchy.

The functional importance of these disinhibitory circuit motifs is evident during learning, where transient reductions in the activity of PV+ interneurons are observed (Gambino and Holtmaat, 2012; Kuhlman et al., 2013; Wolff et al., 2014). Interestingly, transient inhibition of PV+ cells (i.e. disinhibition) also occurs in rodent dorsal medial prefrontal cortex (dmPFC) during exposure to a conditioned stimulus that triggers context-dependent memory retrieval (Courtin et al., 2014). Optogenetic inhibition of these dmPFC PV+ interneurons elicits retrieval of a fear response, suggesting that transient inhibition of selective PV+ interneurons is sufficient for memory recall (Courtin et al., 2014). In humans, parallel signatures of these disinhibitory effects can be observed when the concentration of GABA in the lateral occipital complex is reduced using brain stimulation, leading to an increase in the expression of neocortical memories (Barron et al., 2016; Koolschijn et al., 2019) (Fig. 2B–E).

Within the cortical microcircuit, the diversity of inhibitory subtypes may therefore provide the necessary infrastructure for two principal modes of processing: one that silences cortical activity patterns via inhibition and another that amplifies cortical activity patterns via disinhibition. This perspective is supported by *in silico* simulations, which show that the weighted difference in inputs received by VIP+ neurons versus other inhibitory subtypes may determine the mode of cortical processing (Hertaeg and Sprekeler, 2018; Wong and Wang, 2006). These simulations suggest that descending projections received by the cortical microcircuit can determine the processing mode by varying the weighted input to different interneuron subtypes. Optogenetic manipulations in rodents further corroborate this proposal: when descending projections from cingulate cortex to V1 are routed via SOM+ and PV+ interneurons, V1 pyramidal cells are inhibited. However, when these descending projections are routed via VIP+ interneurons, this inhibition is overwritten via disinhibition, selectively enhancing the response in V1 pyramidal cells (Zhang et al., 2014). Afferents from higher-level areas such as frontal cortex can therefore evoke localised inhibition or disinhibition in lower-level cortical areas, depending on the routing of inhibition within the cortical microcircuit. In an analogous manner, unitary representations in the hippocampus may also have the capacity to evoke decreases or increases in neocortical activity. Optogenetic tools now provide a means to directly test this hypothesis, to characterise and establish the functional significance of hippocampal projections to neocortical regions.

### 1.6 Differential processing modes: neuromodulation and precision

The diversity of interneurons within the cortical microcircuit appears to allow a single circuit motif to have two complementary functions (Fig. 3). When direct descending projections target PV+ or SOM+ interneurons, cortical representations are inhibited, providing a means for descending predictions to suppress or explain away prediction errors in lower levels of the cortical hierarchy. Alternatively, descending predictions can be routed via a disinhibitory pathway, where VIP+ interneurons target PV+ and SOM+ interneurons, to release excitatory pyramidal cells from inhibition. This facilitatory effect provides a mechanism for *selective* neocortical reinstatement that underlies memory recall. In terms of predictive coding, this selection corresponds to the same sort of process underlying attentional selection via the selective increase in the precision of particular prediction errors – based on so-called second-order predictions (Kanai et al., 2015); i.e., predictions of precision. Concurrent, second-order predictions (of precision) can selectively modulate the gain of prediction error units, thereby evincing a form of representational sharpening (Kersten et al., 2004; Kok et al., 2012a; Murray et al., 2002). Notably, in predictive coding, explicit changes in predictability are not necessary for predictions of precision to change, as both first and second-order predictions of content and context are continuously updated with learning (Garrido et al., 2009b; Moran et al., 2013).

Increasing evidence suggests that neuromodulation may encode precision (Friston, 2008). Neuromodulators increase the gain of cortical circuits by shifting the balance between excitation and inhibition (EI) via decreases in inhibition (Alkondon et al., 2000; Froemke, 2015). For example, application of acetylcholine during whole-cell recording in rat visual cortex leads to attenuation of the inhibitory post-synaptic currents received by pyramidal cells (Fu et al., 2014; Pfeffer et al., 2013; Xiang et al., 1998). Similarly, when pairing a visual stimulus with *in-vivo* application of a cholinergic agonist, neuronal responses in V1 increase, suggesting that application of acetylcholine mimics the effects of selective attention on V1 activity (Herrero et al., 2008; Kang et al., 2014). Interestingly, this acetylcholine dependent increase in cortical gain is mediated via disinhibition as inputs from the basal forebrain, the major source of acetylcholine for cortex, target cortical interneurons considered responsible for disinhibition, namely VIP+ interneurons (Alitto and Dan, 2013; Fu et al., 2014) and NDNF interneurons in layer 1 (Poorthuis et al., 2018). Acetylcholine can therefore directly affect cortical gain by weighting the descending inputs received by different interneuron subtypes (Fig. 3).

Evidence in humans further suggests that acetylcholine mediates precision control. When humans are given a cholinesterase inhibitor to boost tonic levels of acetylcholine, cortical responses to unexpected or ‘deviant’ stimuli are enhanced (Moran et al., 2013). Applying biophysically plausible models to this data formalises the role of acetylcholine in modulating gain within the cortical microcircuit (Moran et al., 2013). In this manner, cholinergic inputs to the cortical microcircuit may reflect the precision of representations, which can be formalised as the predicted precision of prediction errors.

By influencing the precision ascribed to prediction errors, neuromodulators may determine the nature of hippocampal-neocortical interactions. But what mediates neuromodulator release? Intriguingly, the residual prediction error signals that ascend the cortical hierarchy – and generate a mismatch signal in the hippocampus – may determine cholinergic tone, which in turn affects both the hippocampal processing mode (Hasselmo, 2006; Hasselmo and Schnell, 1994) and ensuing hippocampal-neocortical interactions (Lisman and Grace, 2005). In rodents, cholinergic terminals co-transmit acetylcholine and GABA, two neurotransmitters that influence whether the hippocampal processing mode is dominated by SWRs (low cholinergic tone) that support episodic recall (Vandecasteele et al., 2014), or by theta rhythms (high cholinergic tone) that support either predictive activity or promote plasticity to facilitate learning of new information (Hasselmo, 2006; Lisman and Grace, 2005). Thus, hippocampal regulation of acetylcholine – together with the accompanying feedback loop – may exercise precision control to set the relative weighting of inhibitory and disinhibitory routing within the cortical microcircuit. Notably, similar mechanisms have also been proposed for other neuromodulators such as dopamine (Lisman and Grace, 2005) and norepinephrine (Vinogradova, 2001).

It is worth noting that – beyond adaptive gain control exerted by classical neuromodulators such as acetylcholine, dopamine, and norepinephrine – precision control may also be mediated by NMDA receptors (NMDARs). These two precision control mechanisms may differentially affect predictions of stimulus content (‘what’ predictions) and its timing (‘when’ predictions). For example, biophysical modelling of stimulus-evoked activity in human sensory cortex suggests that precision of ‘when’ predictions is best explained by classical neuromodulation of cortical gain (e.g., cholinergic and dopaminergic mechanisms), while precision of ‘what’ predictions is better explained by NMDAR-dependent plasticity in sensory regions (Auksztulewicz et al., 2018) (Fig. 2F). Crucially, voltage-dependent NMDARs are particularly abundant on PV+ interneurons (Cornford et al., 2019) that we associate with both the inhibitory and disinhibitory cortical microcircuit pathway. The emerging picture therefore suggests that a particular set of descending (precision predicting) projections modulate disinhibition to optimally regulate neocortical balance between excitation and inhibition, either di-synaptically via VIP+ interneurons that are equipped with nicotinic receptors, or directly via NMDARs on fast-spiking PV+ interneurons (Fig. 3).

In short, we propose that neuromodulators, such as acetylcholine, but also NMDAR mediated neuromodulation, may help determine whether descending projections that originate in the hippocampus have an inhibitory or disinhibitory effect on cortical microcircuits (Fig. 3). The latitude for both driving and modulatory effects of descending projections on neocortical pyramidal cells may provide the necessary machinery for the hippocampus to play a dual role of generating (first-order) predictions and indexing memory via (second-order) predictions of precision. Notably, global neuromodulatory transmitter systems may determine the context (i.e., online versus off-line) of these complementary roles.

### 1.7 Updating a Bayesian model and structure learning

This formulation implicitly extends the predictive coding framework to suggest that precision also mediates cortical excitability when activity patterns *are reinstated off-line*, during memory recall. Thus, high precision increases the gain regardless of whether the cortical circuit receives unexpected input that generates prediction errors *online*, or reinstates activity patterns *offline*. This leads to the prediction that within the cortical microcircuit, cortical reinstatement manifests in exactly the same way as during the predictive processing of the sensorium. Computationally, this seems a natural generalisation of hierarchical inference processes associated with imagination and dreaming (Hinton et al., 1995; Hobson and Friston, 2012). In this setting, predictive coding hierarchies are released from sensory constraints by neuromodulatory suppression of low-level sensory precision; particularly involving cholinergic and noradrenergic neurotransmitter systems (Calvo et al., 1992; Stickgold and Hobson, 1995). This suppression is coupled with a relative increase in precision of prediction errors higher in the processing hierarchy (thought to be mediated by cholinergic afferents) that enable fictive, generative processes and preclude updating by prediction errors ascending from lower (e.g., sensory) levels. This is exactly the scenario that would be necessary for hippocampal-dependent recall and imagery. To illustrate this intuitively, we typically find it easier to imagine with our eyes closed, when the signal-to-noise of the sensory input (or sensory precision) is attenuated. By closing our eyes, we effectively tip the weighting of precision in favour of top-down, prior beliefs, such that perceptual content is biased towards autonomous (e.g., recalled) experience rather than sensory evidence.

Having characterised memory recall within a predictive coding framework, the computational parallels between memory recall and prediction error signals become increasingly apparent. Prediction error signals arise through a mismatch between descending inhibitory predictions and ascending excitatory sensory input that manifest as prediction errors as they pass through the processing hierarchy. These prediction error signals are used to update representations to ensure better estimates for future experience. If cortical reinstatement during memory recall engages the same neuronal mechanisms, then, like prediction error signals, memory recall may provide a training signal that can be used to update our generative models. This is precisely the computational strategy used in machine learning schemes such as the wake-sleep algorithm (Hinton et al., 1995).

It may seem odd to suggest that recalling old experiences – or constructing new ones – can improve a model in the absence of new sensory evidence. However, this is exactly how many computational schemes minimize statistical complexity and preclude overfitting; i.e., removing redundant components to ensure the model generalizes to new data. Technically, this can be viewed as a process of Bayesian model learning, based upon the maximization of Bayesian model evidence. Mathematically, model evidence is the difference between the *accuracy* and *complexity* of a model’s predictions of (sensory) data (see Box 1). This means that model evidence can be increased by reducing complexity in the absence of any new data. In summary, the generation of fictive (offline) prediction errors is an essential part of machine learning schemes (Hinton et al., 1995) and has been proposed as the basis of synaptic homoeostasis (Gilestro et al., 2009; Tononi and Cirelli, 2006) These purely theoretical considerations seem to be particularly prescient for the role of the hippocampus in sleep (Buckner, 2010; Buzsaki, 1998). Furthermore, they speak to the mechanisms that may underwrite more general structure learning in finessing our generative models of the world (Gershman, 2017; Tenenbaum et al., 2011; Tervo et al., 2016).

Box 1 **Glossary of terms**

**Memory index:** During memory recall, the hippocampal index facilitates neocortical reinstatement of selective activity patterns to recapitulate previous experience. The memory index thus represents a unique identifier for experiential events represented across distributed neocortical networks.

**Reinstatement:** A neural activity pattern observed during memory recall that was present during a previous experience.

**System-level consolidation theory:** Proposes that episodic memories are stored in progressively strengthened cortico-cortical connections that become independent of the hippocampal memory trace with time.

**Multiple-Trace theory:** Each reactivation of an episodic memory results in a different trace in the hippocampus; hippocampal ensembles are always involved in storage and retrieval of episodic information.

**Generative Model:** Generates predictions about incoming sensory input. To better predict future sensory input, the generative model is updated by the mismatch between the generated prediction and the received sensory input, otherwise termed the prediction error signal.

**Prediction error:** A quantity used in predictive coding to denote the difference between an observation, point estimate, or sensory input and its predicted value. Prediction error signals carry the only information yet to be explained.

**Precision:** reflects the reliability or inverse variability of a variable. The precision of a prediction error describes the reliability of the prediction error; i.e., the weight afforded to a prediction error when revising or updating state estimates or representations (a.k.a. Bayesian belief updating).

**Disinhibition:** involves relieving excitatory neurons from ongoing inhibition to favour excitation and thereby enhance their responsiveness. Disinhibition may be achieved by inhibiting inhibitory interneurons that directly target excitatory cells.

**PV+**: Parvalbumin positive interneurons are the largest category of inhibitory cells. They are found throughout cortical layers 2–6, are typically fast-spiking and predominantly target the perisomatic region of excitatory principal cells (and other PV+ cells).

**SOM+:** Somatostatin positive interneurons are a diverse subset of inhibitory cells that predominantly target the dendrites of excitatory principal cells.

**VIP+:** Vasoactive intestinal peptide positive interneurons primarily synapse onto other GABAergic interneurons, providing inhibition to PV+ and particularly SOM+ interneurons. VIP+ interneurons thus play a role in disinhibiting cortical circuits.

**Bayesian beliefs**: non-propositional probabilistic beliefs that correspond to posterior probability distributions (whose sufficient statistics are) encoded by neuronal activity and connectivity.

**Bayesian belief updating**: the process of updating (the sufficient statistics) of Bayesian beliefs about the causes of (sensory) data. There are many particular schemes that implement Bayesian belief updating; for example, belief propagation, variational message passing, and predictive coding have all been proposed as formal descriptions of neuronal dynamics or message passing.

**Bayesian model evidence:** also known as integrated or marginal likelihood is the likelihood that some data were generated by a particular model. It can always be decomposed into accuracy (the probability of the data expected under some model parameters) and complexity (the number of parameters needed to provide an accurate explanation of the data).

**Bayesian model learning**: a.k.a. Bayesian model selection, updating or structure learning. The process of updating the generative model that underwrites Bayesian belief updating. This would normally involve removing redundant parameters; e.g., pruning redundant synaptic connections. This is a slower process that tries to optimise Bayesian belief updating by a generative model that is sufficiently complex to provide an accurate account of (sensory) data as simply as possible.

Alt-text: Box 1

### 1.8 Conclusion

In this hypothesis piece, we asked how the hippocampus furnishes both an index for cortical memory recall, and predictions of cortical representations during sensory experience. We use a predictive coding framework to explore how this dual aspect hippocampal function may have opposing effects on cortical processing, despite a seemingly unitary hippocampal code. To dissolve this dialectic, we use theoretical constraints and empirical evidence to characterise hippocampal-neocortical interactions, and generate a number of testable predictions (Box 2). The picture that emerges suggests a special role for neocortical inhibitory interneurons in determining hippocampal-neocortical interactions. Crucially, the diversity of inhibitory interneurons appears to mediate both direct (driving) inhibition and disinhibitory (modulatory) mechanisms, which allows for both inhibitory (prediction) and facilitatory (recall) hippocampal-neocortical interactions. Furthermore, we propose that precision, as defined in predictive coding and implemented by neuromodulation, provides the computational formalism to disambiguate these two modes of constructive processing. Finally, within this framework, cortical reinstatement during memory recall may underwrite our remarkable capacity to improve generative models of the world; even in the absence of new data. Recent advances in genetic techniques provide an exciting opportunity to test these predictions, which sit at the heart of learning, memory and sentience.

Box 2 **Testable Predictions**

1 During ongoing sensory experience, descending predictions generated by the hippocampus should elicit cortical inhibition. This may be achieved either by (1) excitatory hippocampal projections that target **local** inhibitory interneurons in the neocortical circuits; (2) long-range GABAergic projections from hippocampus to excitatory pyramidal cells in neocortex.
2 During memory recall, the hippocampus should reinstate neocortical activity patterns via a disinhibitory mechanism that involves either recruiting VIP+ neocortical interneurons, or NMDA receptor dependent modulation of fast-spiking PV+ neocortical interneurons.
3 Memory recall should be accompanied by an increase in precision of prediction errors high in the processing hierarchy – that may be sensitive to cholinergic manipulations. While *online* prediction errors can be measured as sensory mismatch responses, *offline* (fictive) prediction errors can be indirectly measured as neural responses evoked by noise bursts presented during memory recall (Wolff et al., 2017). Increasing the level of acetylcholine using Galantamine (a cholinesterase inhibitor) should increase the amplitude of these evoked responses at late latencies and in extrasensory regions. These amplitude modulations should be explained by increased gain of pyramidal cells in extrasensory regions (Moran et al., 2013).
4 Within the cortical microcircuit, two distinct influences of hippocampal projections should be discernible: via a direct inhibitory or indirect disinhibitory pathway. By identifying neocortical targets of descending projections from the hippocampus in mice (Testable Prediction 1), optogenetic manipulations may be able to characterise the effect of descending hippocampal projections on different inhibitory subtypes, with and without stimulation of cholinergic afferents.
5 If memory recall provides a fictive prediction error signal, offline reinstatement in low levels of the cortical hierarchy should update representations in higher levels of the cortical hierarchy, including in the hippocampus. In other words, the fictive prediction error signal should provide a training signal that is used to update the generative model. By combining representational fMRI with a careful experimental design, it may be possible to test this prediction by asking how memory recall in lower levels of the cortical hierarchy updates representations in higher levels of the cortical hierarchy, including in the hippocampus.

Alt-text: Box 2

## Funding

H.C.B. was supported by a Junior Research Fellowship from Merton College (University of Oxford) and the Medical Research Council (MRC) UK (MC_UU_12024/3 and MC_UU_00003/4); K.F. was supported by the 10.13039/100010269Wellcome Trust (Ref: 088130/Z/09/Z). R.A. is supported by the European Commission’s Marie Skłodowska-Curie Global Fellowship (750459).

## Footnotes

1 In this treatment, we are not concerned with how signed prediction errors are encoded neuronally. Although this is an interesting debate (Keller and Mrsic-Flogel, 2018), the encoding of signed prediction errors (required in predictive coding) with non-negative firing rates can be accounted for – in terms of neuronal implementation – by noting that signed prediction errors could be reported by the logarithm of unsigned firing rates – or deviations from baseline firing. In this treatment, we are concerned with the fluctuations in prediction errors (whether they are signed or unsigned).
